# Polygenic risk score assessment of LDL-Cholesterol levels and progression to severe hypercholesterolemia and statin treatment; a longitudinal study from a large middle eastern biobank

**DOI:** 10.1016/j.ajpc.2026.101768

**Published:** 2026-07-24

**Authors:** Umm-Kulthum Ismail Umlai, Salman M Toor, Usama Aliyu, Shaban Mohammed, Nadeem Qureshi, Moza S.H. Al Hail, Mohammed Gomaa, Amin Jayyousi, Jassim M. Al Suwaidi, Omar M.E. Albagha

**Affiliations:** a College of Health and Life Sciences (CHLS), Hamad Bin Khalifa University (HBKU), Qatar Foundation (QF), P.O. Box 34110, Doha, Qatar; b Diabetes Research Center, Qatar Biomedical Research Institute (QBRI), Hamad Bin Khalifa University (HBKU), Qatar Foundation (QF), P.O. Box 34110, Doha, Qatar; c Department of Pharmacy, Hamad Medical Corporation, P.O. Box 3050, Doha, Qatar; d Primary Care Stratified Medicine Research Group, Centre for Academic Primary Care, School of Medicine, University of Nottingham, Nottingham, United Kingdom; e Adult Cardiology, Heart Hospital, Hamad Medical Corporation (HMC), Doha, Qatar; f Department of Diabetes, Hamad Medical Corporation (HMC), Doha, Qatar

**Keywords:** Cholesterol, GWAS, Hypercholesterolemia, LDL-C, PGS, Polygenic risk score

## 1 Introduction

Hypercholesterolemia (HC), particularly elevated low-density lipoprotein cholesterol (LDL-C), is a major modifiable risk factor for atherosclerotic cardiovascular disease and affects approximately 20% of the global population [1]. LDL-C is influenced by monogenic, polygenic, lifestyle and environmental factors. Over 120 LDL-C polygenic scores (PGS) are listed in the PGS Catalog [2], but most were developed in European or predominantly European datasets, and their performance can be reduced in other ancestries because of differences in linkage disequilibrium, allele frequencies and effect sizes [3]. Moreover, existing LDL-C PGS have largely been evaluated using cross-sectional lipid traits or case-control definitions rather than longitudinal progression to clinically severe hypercholesterolemia. In previous work in the Qatar Biobank (QBB), multi-ancestry-derived PGS showed the strongest performance for LDL-C prediction [4]. Here, we integrated 11 published LDL-C PGS into a composite PGS (cPGS) and evaluated its association with treatment-corrected LDL-C, progression to severe hypercholesterolemia and statin treatment in the longitudinal QBB cohort.

## 2 Materials and methods

### 2.1 Study cohort

The study included 13,695 native Qatari participants from QBB with whole-genome sequencing and phenotypic data. Whole-genome sequencing and serum LDL-C measurements were described previously [4]. LDL-C values were corrected for cholesterol-lowering treatment as described previously [5]. Severe hypercholesterolemia (Severe-HC) was defined as corrected LDL-C ≥ 4.9 mmol/L while the statin-treatment group (Statin-Tx) comprised participants on treatment and had corrected LDL-C < 4.9 mmol/L. Participants not on statins and with LDL-C < 4.9 mmol/L were considered without severe hypercholesterolemia. The study was approved by the Institutional Review Boards of QBB (E-2020-QF-QBB-RES-ACC-0154–0133) and Hamad Bin Khalifa University (QBRI-IRB 2021–03–081), and all participants provided written informed consent.

### 2.2 Polygenic scores assessment and statistical analysis

Eleven published LDL-C PGS from diverse populations were selected as previously described [2,4,6]. PGS were calculated for all participants and normalized within each cohort using PLINKv2.0. In the training cohort, LDL-C and the 11 PGS were used in an elastic net model to derive the cPGS. In the validation cohort, linear regression assessed association between PGS and treatment-corrected LDL-C and reported adjusted R^2^ and incremental adjusted R^2^ over clinical covariates. Participants were stratified into quintiles (Q1 to Q5) of cPGS genetic risk. Cox proportional hazards models in the test cohort estimated hazard ratios (HR) per SD increase in PGS and by cPGS quintile. Discrimination was assessed using the C-index, and incremental improvement was quantified as ΔC-index and Net Reclassification Improvement (NRI) using bootstrapping (2,000 resamples). Sensitivity analyses were performed after adding baseline LDL-C to the model, and in age- and BMI-matched groups using stratified Cox regression. Continuous variables are reported as mean (SD), and categorical variables as n (%). Welch's *t*-test and chi-square tests were used to compare groups. Analyses were conducted in R v4.1.4, with *P* < 0.05 considered significant.

## 3 Results

### 3.1 Characteristics of the study cohort

The study design is depicted in Fig. 1A, while test cohort characteristics are presented in Fig. 1B During approximately six years of follow-up, 117 participants progressed to severe hypercholesterolemia, 140 initiated statin treatment and 1,846 remained free of severe hypercholesterolemia. Age, BMI and LDL-C were higher among progressors than non-progressors; both at baseline and follow-up.

**Fig. 1:**
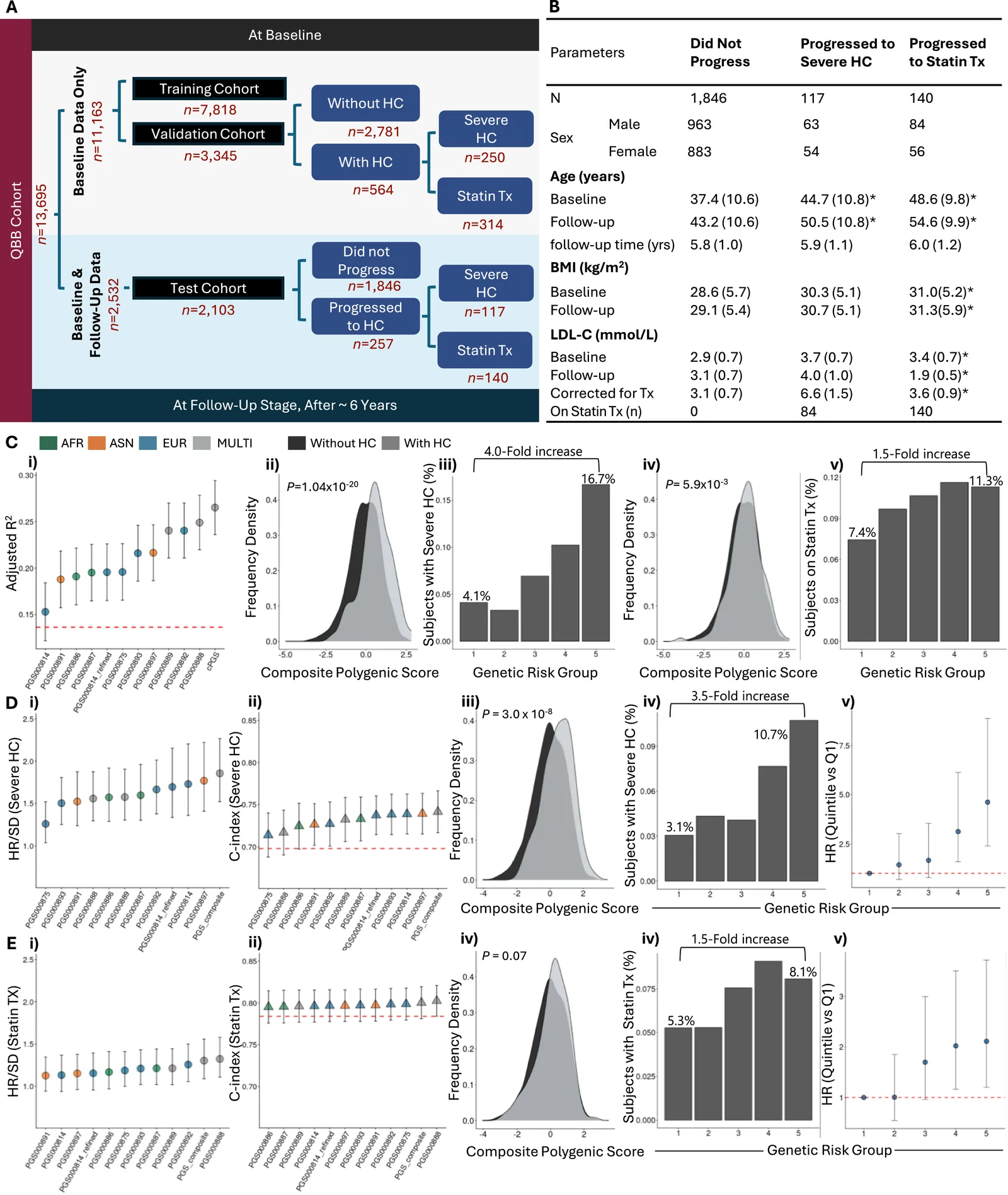
(A) Study design. The study included 13,695 native Qatari participants from Qatar biobank (QBB) with whole-genome sequencing and phenotypic data. Participants with baseline-only data were randomly partitioned into 70% training (*n* = 7,818) and 30% validation (*n* = 3,345) cohorts. Participants with baseline and follow-up data formed the longitudinal test cohort (*n* = 2,532); after excluding individuals with incomplete data (*n* = 18) and those with hypercholesterolemia at baseline (corrected LDL-C ≥ 4.9 mmol/L or on statin treatment; *n* = 411), 2,103 remained in the test cohort. Validation and test cohort were further stratified by severe HC (defined as LDL-C levels ≥ 4.9 mmol/L after correction for statin treatment), Statin Tx (defined as subjects on statin treatment and their LDL-C levels < 4.9 mmol/L), and without severe HC (subjects not on statin treatment and their LDL-C levels < 4.9mmol/L). (B) Test cohort characteristics (*n* = 2,103) Over ∼ 6 years follow-up time, 117 subjects progressed to Severe HC, 140 progressed to incident Statin-Tx, and 1,846 remained without severe HC. Continuous values shown as mean (SD). *P-values <0.05, Welch’s *t*-test compared to non-progressors. (C) PGS performance assessment in the validation cohort, (i) Performance of individual PGS predicting corrected LDL-C using linear regression models (including age, sex, PGS, and genetic principal components) in the validation cohort; all *P* < 0.001 except for PGS000814; Error bars are 95% CI; red dashed line represents adjusted R² of the base model including age and sex. (ii) frequency density of cPGS comparing those with severe HC (*n* = 250) to those without severe HC (*n* = 2,781); (iii) proportion of subjects with severe HC per cPGS quintile; (iv) frequency density of cPGS comparing those with Statin Tx (*n* = 314) to those without severe HC (*n* = 2,781); (v) Proportion of subjects with Statin Tx per cPGS quintile. (D) PGS performance predicting progression to severe HC in the test cohort; (i) Hazards ratio (HR) per standard deviation of PGS, (ii) Concordance index (C-index) for models including age, sex, BMI, PGS, and genetic principal components; red dashed line depicts C-index for the basic clinical model including age, sex, and BMI; Error bars are 95% CI for HR and standard error for C-index. (iii) frequency density of cPGS comparing those with severe HC (*n* = 117) to those without (*n* = 1,846); (iv) proportion of subjects with severe HC per cPGS quintile; (v) Hazards ratio (HR) per cPGS quintile compared to the lowest quintile. (E) PGS performance predicting progression to Statin Tx in the test cohort; (i) Hazards ratio (HR) per standard deviation of PGS, (ii) Concordance index (C-index) for models including age, sex, BMI, PGS, and genetic principal components; red dashed line depicts C-index for the basic clinical model including age, sex, and BMI; (iii) frequency density of cPGS comparing those with Statin Tx (*n* = 140) to those without severe (*n* = 1,846); (iv) proportion of subjects with Statin Tx per cPGS quintile; (v) Hazards ratio (HR) per cPGS quintile compared to the lowest quintile.

### 3.2 Performance of PGS in predicting LDL-C levels and hypercholesterolemia in the validation cohort

In the validation cohort, most PGS predicted corrected LDL-C, but cPGS (which included 1,102,026 variants) had the largest incremental improvement when added to the model that included age and sex (Δ adjusted R^2^ = 0.1289; *P* < 0.001; Fig. 1C-i). Participants with severe hypercholesterolemia had substantially higher cPGS than those without hypercholesterolemia (0.58 versus −0.06; *P* < 0.001; Fig. 1C-ii), and the proportion with severe hypercholesterolemia increased approximately 4-fold from the lowest (Q1) to highest (Q5) cPGS quintile (Fig. 1C-iii). The statin-treatment group showed a smaller but significant increase in cPGS (0.098 versus −0.06; *P* < 0.01; Fig. 1C-iv), with ∼1.5-fold increase in the highest versus lowest cPGS quintile (Fig. 1C-v).

### 3.3 Performance of PGS in predicting progression to severe hypercholesterolemia or incident statin treatment in the test cohort

In the longitudinal test cohort, cPGS predicted progression to severe hypercholesterolemia (HR/SD = 1.86; 95%CI 1.52–2.27; *P* < 0.001; Fig. 1D-i) and improved discrimination beyond age, sex, and BMI (ΔC-index = 0.043; NRI = 0.325; *P* < 0.05; Fig. 1D-ii). Severe-hypercholesterolemia progressors had higher cPGS than non-progressors (0.47 versus −0.04; *P* < 0.001; Fig. 1D-iii). The highest cPGS quintile had a 3.5-fold higher proportion of severe hypercholesterolemia cases and an HR of 4.62 (95% CI 2.40–8.89; *P* < 0.001) compared with the lowest quintile (Fig. 1D-v).

Prediction of incident statin treatment was weaker (cPGS HR/SD = 1.30; 95%CI 1.09–1.56; *P* < 0.01, ΔC-index = 0.016; *P* = 0.10; Fig. 1Ei). After adding baseline LDL-C to prediction models, cPGS remained associated with severe hypercholesterolemia (HR/SD = 1.38; 95%CI 1.11–1.71; *P* < 0.01), but the incremental improvement was not significant (ΔC-index = 0.008; *P* = 0.33; NRI = 0.272; *P* = 0.12), and no PGS predicted incident statin treatment.

In age- and BMI-matched analyses, cPGS remained associated with progression to severe hypercholesterolemia and showed greater incremental discrimination (HR/SD = 1.99; 95%CI 1.48–2.68; ΔC-index = 0.230; NRI = 0.494; *P* < 0.005). Similar, although less pronounced, findings were observed for incident statin treatment (HR/SD = 1.53; 95%CI 1.18–2.00; ΔC-index = 0.100; NRI=0.582; *P* < 0.005).

## 4 Discussion

We developed a cPGS and evaluated its performance in predicting corrected LDL-C levels, progression to severe hypercholesterolemia, and incident statin treatment. Most existing LDL-C polygenic scores have been developed using cross-sectional lipid measurements or case-control studies and have rarely been evaluated against longitudinal disease progression.

In this study, cPGS predicted future progression to severe hypercholesterolemia; the highest cPGS quintile had a 3.5-fold higher proportion of severe hypercholesterolemia cases and an HR of 4.6 compared with the lowest quintile. Importantly, the cPGS provided significant incremental predictive value beyond conventional clinical variables, including age, sex, and BMI, highlighting its potential utility for early risk stratification.

The performance of cPGS was weaker for incident statin treatment, likely because statin initiation also reflects clinical decision-making, healthcare access, comorbidities and patient preferences. Interestingly, the incremental predictive value of cPGS increased considerably in age- and BMI-matched analyses which suggests that cPGS captures risk information that is largely independent of age and adiposity and may be particularly useful for identifying individuals at increased risk despite otherwise similar clinical profiles.

Notably, when baseline LDL-C levels were incorporated into the prediction model, cPGS remained associated with progression to severe hypercholesterolemia; however, the incremental improvement in discrimination was no longer significant. This observation suggests that while genetic risk contributes to long-term susceptibility, measured LDL-C remains a strong predictor once available. However, the primary objective of a polygenic score is to identify individuals at increased risk before clinically significant LDL-C elevation occurs and before severe hypercholesterolemia develops.

In this study, cPGS predicted future progression to severe hypercholesterolemia, supporting its potential role for earlier risk stratification before traditional clinical risk factors become strongly predictive. Following external validation, such approaches may complement conventional risk assessment strategies and facilitate earlier identification of individuals who could benefit from preventive intervention.

Strengths include the large population-based cohort, whole-genome sequencing, longitudinal follow-up, independent training, validation and test datasets, and treatment-corrected LDL-C. Limitations include the absence of external validation, analysis based on single follow-up visit, and potential residual effects of statin treatment. To our knowledge, this is the first longitudinal evaluations of LDL-C PGS for severe hypercholesterolemia progression in a Middle Eastern population.

## Funding

This work was supported by a grant from the Qatar National Research Fund (QNRF) grant awarded to OMEA (PPM 03-0324-190038).

## CRediT authorship contribution statement

**Umm-Kulthum Ismail Umlai:** Writing – original draft, Investigation, Formal analysis, Data curation. **Salman M Toor:** Writing – review & editing, Methodology, Investigation. **Usama Aliyu:** Writing – review & editing, Methodology, Investigation. **Shaban Mohammed:** Resources. **Nadeem Qureshi:** Resources. **Moza S.H. Al Hail:** Resources. **Mohammed Gomaa:** Resources. **Amin Jayyousi:** Resources. **Jassim M. Al Suwaidi:** Resources. **Omar M.E. Albagha:** Writing – review & editing, Supervision, Investigation, Funding acquisition, Formal analysis, Conceptualization.

## Declaration of competing interest

The authors declare that they have no known competing financial interests or personal relationships that could have appeared to influence the work reported in this paper.

## Data Availability

The data analyzed in this study can be obtained through an ISO-certified protocol. Requests to access these datasets are subject to approval by the Institutional Review Board of the Qatar Precision Health Institute (QPHI), Qatar Foundation, Doha, Qatar and should be directed to https://www.qphi.org.qa/research/how-to-apply.
